# Effective Primary Prevention of Atopic Dermatitis in High-Risk Neonates via Moisturizer Application: Protocol for a Randomized, Blinded, Parallel, Three-Group, Phase II Trial (PAF Study)

**DOI:** 10.3389/falgy.2022.862620

**Published:** 2022-04-04

**Authors:** Yusuke Inuzuka, Kiwako Yamamoto-Hanada, Kyongsun Pak, Takekazu Miyoshi, Tohru Kobayashi, Yukihiro Ohya

**Affiliations:** ^1^Allergy Center, National Center for Child Health and Development, Tokyo, Japan; ^2^Division of Biostatistics, Clinical Research Center, National Center for Child Health and Development, Tokyo, Japan; ^3^Department of Clinical Research Promotion, Clinical Research Center, National Center for Child Health and Development, Tokyo, Japan; ^4^Department of Data Science, Clinical Research Center, National Center for Child Health and Development, Tokyo, Japan

**Keywords:** atopic dermatitis, moisturizer, neonate, randomized, controlled trial

## Abstract

**Background:**

Atopic dermatitis (AD) is a chronic and inflammatory skin disease that causes health-related burdens associated with pruritus and poor quality of life. Our previous study demonstrated that moisturizer (2e) application has a primary preventive effect on AD. However, this effect was not observed in recent randomized control trials. Thus, the ideal moisturizer type and application frequency for preventing AD development in infants remains unclear. We hypothesize that twice daily application of moisturizer is more effective than once daily application. We predict that applying sufficient amounts of high-quality moisturizer may be effective for preventing AD development in neonates and infants. Here, we describe a protocol for comparing the efficacy of twice daily and once daily application of Fam's Baby™ moisturizer and once daily application of 2e moisturizer for preventing AD in neonates.

**Methods:**

This study is a single-center, three-parallel group, assessor-blind, superiority, individually randomized, controlled, phase II trial. Sixty newborns with at least one parent or sibling who has had AD is randomly assigned to application of Fam's Baby twice daily, Fam's Baby once daily, or 2e once daily in a 1:1:1 ratio until 32 weeks old. The primary outcome is the time to the first onset of AD during administration of the moisturizer.

**Discussion:**

This is the first phase II randomized, controlled trial in Japan to estimate how effective the twice daily or once daily application of Fam's Baby moisturizer is in preventing AD compared to the once daily application of 2e moisturizer. In this study, we will use 2e once daily as a control to confirm the efficacy for primary prevention of AD as found in our previous trial. Based on the results of this study, we hope to conduct a phase III study to determine the optimal method for preventing AD via moisturizer application. Evaluation of application of moisturizers for preventing AD in this study is expected to contribute to a reduction in the prevalence of AD and a reduction in health care costs.

**Trial registration:**

Japan Registry of Clinical Trials (jRCT); ID: jRCTs031200070.

## Introduction

### Background and Rationale

Atopic dermatitis (AD) is a chronic and inflammatory skin disease that commonly affects young children. AD causes health-related burdens associated with pruritus and poor quality of life (1). A systematic review and meta-analysis showed that parental history of AD increased the risk of AD in the offspring [pooled odds ratio: 3.30, 95% confidence interval (CI): 2.45–4.42] (2). Genetically susceptible individuals have a higher risk of developing AD.

According to a global epidemiological study conducted between 1994 and 1996, the prevalence of AD as reported by parents in a questionnaire was ~7% among school-age children (3). The prevalence of AD as confirmed by a physician's examination in Japan is 12.8% in children aged 4 months old, 9.8% at 1.5 years old, and 13.2% at 3 years old (1, 4). In a previous cohort study involving 1,157 children in Tokyo, Japan, 32.3% of children were diagnosed with AD by age nine, and four AD phenotypes were identified (5).

The possibility of percutaneous sensitization via inflammatory skin of AD is established on the basis of Lack's dual-allergen-exposure hypothesis (6). We found that early-onset infant AD was positively associated with later food allergy in a prospective cohort study (7). AD is considered as an origin of atopic march and the first history of atopic manifestations in children (8). Therefore, whether skin care intervention from an earlier stage of life is effective for primary prevention of allergic diseases, such as AD and food allergies, would be interesting to determine in the future.

In 2014, we performed a randomized, controlled trial (RCT) to evaluate whether moisturizer (2e, Shiseido Japan Co., Ltd., Tokyo, Japan) once daily prevents the incidence of AD at 32 weeks of age compared with a control group in which Vaseline was applied as required from birth (9). This study was a definitive trial, and 118 neonates participated showed that the cumulative incidence of AD was significantly lower in the 2e application group compared with the control group (hazard ratio: 0.48, 95% CI: 0.27–0.86). Recently, Chalmers et al. (10) reported that application of the moisturizer Doublebase Gel (Dermal Laboratories, Herts, UK) or Diprobase Cream (Bayer, Berks, UK) at least once daily had no preventive effect on AD (adjusted relative risk: 0.95; 95% CI: 0.78–1.16; *p* = 0.61). Similarly, Skjerven et al. (11) conducted a study (2 × 2 factorial design) testing two primary prevention strategies (skin care and early food introduction) and showed that an oil bath and application of a moisturizer to the infant's face (Ceridal cream) at least 5 days per week from 0.5 to 9 months of age was not effective in preventing AD. Therefore, unfortunately, these two large RCTs could not confirm the efficacy of moisturizer application for primary prevention of AD. We speculated that these inconsistent findings might be due to different countries and cultures, different moisturizer types, and inadequate adherence in the two large RCTs compared to the previous RCT that reported a preventive effect. The application of high-quality moisturizers in sufficient amounts may effectively prevent the onset of AD in newborns and infants. The efficacy of applying moisturizer is inconsistent regarding primary prevention of AD based on data from previous RCTs. Therefore, the most effective moisturizer type and application frequency for preventing AD development in infants remains unclear.

Fam's Baby (Fam's Inc., Tokyo, Japan) is a popular commercially available moisturizer in Japan. Fam's Baby contains glycerin, stearic acid, dimethicone, liquefied petroleum gas, polyvinylpyrrolidone, triethanolamine, cetanol, caprylic/capric triglyceride, laureth-2, laureth-21, tocopheryl acetate, and phenoxyethanol. Fam's Baby was demonstrated to protect the skin from allergens in a mouse model (unpublished in-house data). In addition, many consumers who used Fam's Baby commented that the skin is maintained in good condition when using Fam's Baby. We hypothesize that twice daily moisturizer application using Fam's Baby is more effective than once daily application and that a sufficient amount and qualities of moisturizer application is effective for preventing development of AD in neonates and infants.

### Objectives

This study aimed to estimate how effective the twice daily or once daily application of Fam's Baby moisturizer is in preventing AD compared to the once daily application of 2e moisturizer.

### Trial Design

**Figure 1:**
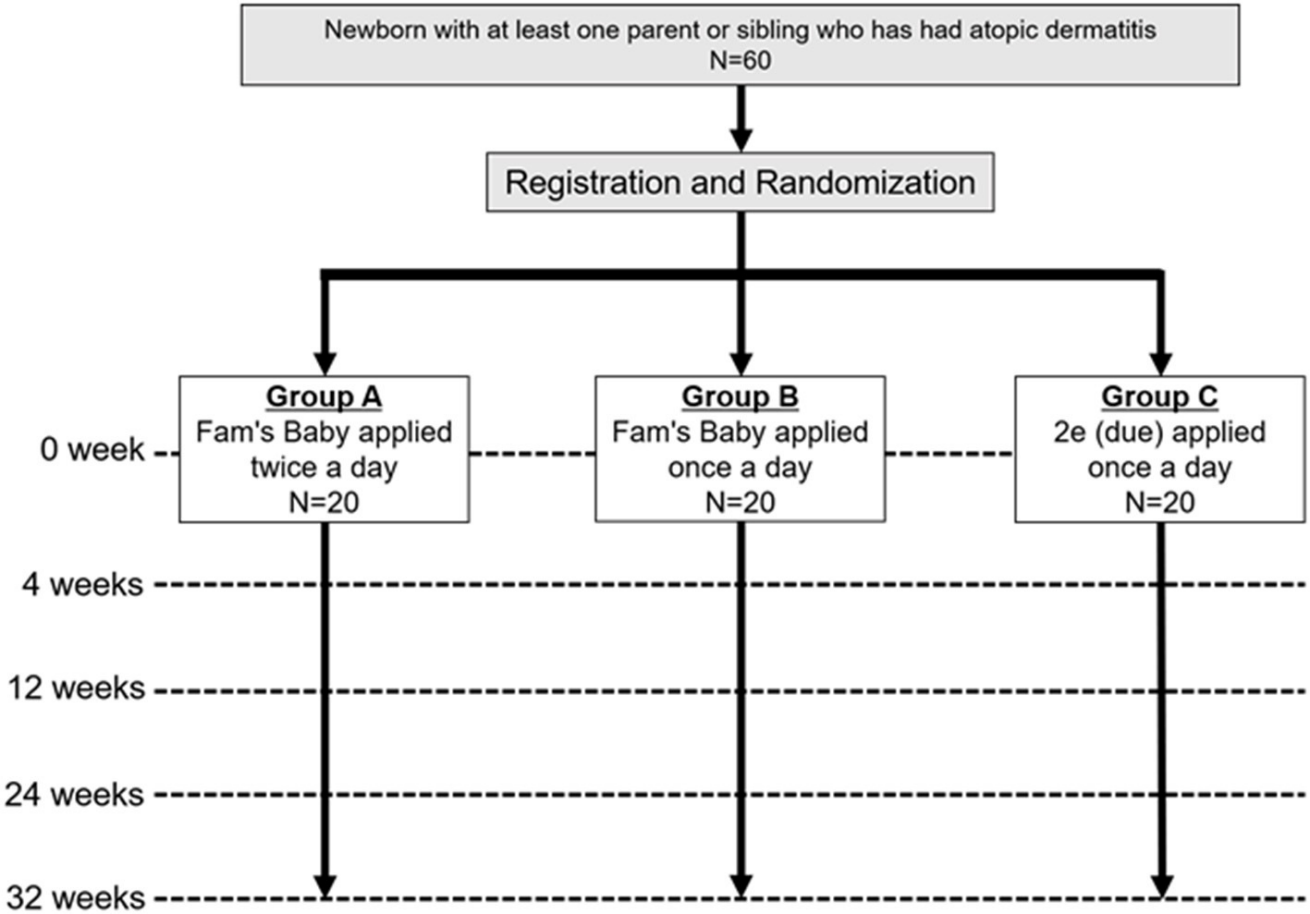
Trial scheme. Newborns with at least one parent or sibling who has had atopic dermatitis will be recruited and enrolled. Participants will be assigned 1:1:1 to groups A, B, and C, and evaluated if they have AD at 4, 12, 24, and 32 weeks for each group.

This study is a single-center, pragmatic, three-parallel group (1:1:1), assessor-blind, superiority, individually, randomized, controlled, phase II trial (Figure 1).

## Methods

### Study Setting

This study will recruit neonates in the National Children's Hospital (National Center for Child Health and Development [NCCHD], Tokyo, Japan).

### Eligibility Criteria

Neonates with a high-risk of developing AD (*N* = 60) who meet all of the following inclusion criteria and none of the exclusion criteria will be enrolled in this study. Physicians will check if parents or siblings have a history of AD based on the self-report.

### Inclusion Criteria

Neonates must meet the following inclusion criteria to be enrolled in the study:

1. Newborns aged younger than 6 days old at recruitment
2. Newborns with at least one parent or sibling who has a past history of AD (high-risk newborn)
3. Newborns whose parents provide written informed consent after receiving an explanation

### Exclusion Criteria

The following exclusion criteria will be used for the study:

1. Newborns using topical medications, such as steroids (excluding the mouth and anal areas)
2. Newborns with skin lesions, such as abnormal keratinization and bullous disease
3. Newborns who are born at <37 weeks in gestation
4. Newborns who are born by cesarean section
5. Multiple births (not singletons)
6. Newborns with serious complications that require long-term hospitalization (e.g., heart malformations, respiratory disorders that require intubation)
7. Newborns whose immediate family plans to move and who may not be able to visit the study site
8. Parents unable to understand Japanese
9. Physicians decide that the neonate is not appropriate for participation in the study

### Interventions

Study physicians will provide guidance to the legal representative (parents) or their family members on how to bathe, how to apply moisturizer, and how to write a diary at the enrollment. These physicians will check the status of entries in the diary for each visit. Participants will be instructed to keep a daily diary to record moisturizer applications, baths, use of combination therapies, occurrence and appearance of rashes, and adverse events. We will instruct not to apply a moisturizer on the visitation day to ensure that the stratum corneum hydration is not affected.

### Intervention in Each Group

1. Group A: Fam's Baby applied twice a day
  • Application site: the whole body, except for the scalp
  • Amount used once: size of a ping-pong ball (0.7 g/piece) with a total of six pieces (total of 4.2 g)
  • Number of applications: twice a day (of the two applications, one was applied after bathing)
  • Application period: until 32 weeks of age

2. Group B: Fam's Baby applied once a day
  • Application site: the whole body, except for the scalp
  • Amount used once: size of a ping-pong ball (0.7 g/piece) with a total of six pieces (total of 4.2 g)
  • Number of applications: once a day after bathing
  • Application period: until 32 weeks of age

3. Group C: 2e applied once a day (9)
  • Application site: the whole body except for the scalp
  • Amount used once: one teaspoonful (4.0 g)
  • Number of applications: once a day after bathing
  • Application period: until 32 weeks of age

### Intervention and Control Groups

The control group, Group C, will apply 2e moisturizer once a day. In our previous RCT, 2e was shown to prevent AD in neonates with a high-risk of developing AD (9). Based on those findings, we recommend moisturizers for this purpose in clinical practice. The efficacy of Fam's Baby moisturizer for preventing AD development has not yet been evaluated but is expected to have a preventive effect. We considered including a group without moisturizer application as a control; however, we recommend moisturizer application as best practice for neonate skincare. Thus, we determined that including a control group without moisturizers is ethically unacceptable and may interfere with recruitment.

### Criteria for Discontinuing or Modifying Allocated Interventions

Study physicians will discontinue the intervention if the research cannot be continued for the following reasons:

1. When AD is diagnosed
2. Parents request to withdraw the neonate from the study or consent is withdrawn
3. When the inclusion criteria are not met or the exclusion criteria are violated after registration
4. When the intervention is difficult to continue because of illness, etc.
5. When participants do not come to the study site owing to moving
6. Discontinuation of the study is deemed appropriate by the study physicians

Study physicians will consider whether to continue the study in the following cases:

1. When important information regarding the quality, safety, and efficacy of the intervention is obtained
2. When completing the study is determined as difficult to complete owing to difficulty in recruiting study participants or frequent dropouts
3. When accepting the intervention is determined as difficult because there is an instruction to change the study plan by the independent data monitoring committee or by the Certified Clinical Research Review Committee

### Strategies to Improve Adherence to Interventions

When recruiting participants, if participants are assigned to groups B and C, we will carefully explain to them that they could not apply the moisturizer more than once, and we will also obtain their consents. We will also give the participants approximately the required amount of moisturizer for each visit, and the amount of moisturizer actually used will be calculated by collecting a container of moisturizer at each visit and by measuring the remaining amount, and the frequency of intervention will be confirmed from the diary written by the parents.

### Relevant Concomitant Care Permitted or Prohibited During the Trial

1. Once a day, the whole body will be washed with soap at the out of bathtub and the soap will be rinsed with water. We will distribute soap to all participants to ensure that the same soap is used.
2. Bath additives will not be used.
3. During the study period, applying other moisturizers, skin topical medicines, and skin application agents is prohibited (excluding the mouth and anal areas).
4. If a skin rash that is not AD appears, physicians will judge whether application of the moisturizer should be interrupted or stopped, and the physicians will start treatment for the skin rash as necessary. If the study intervention is discontinued and treatment improves the condition of the skin, intervention will be resumed. Physicians will record the diagnosis, details of the treatment, and treatment period in an electronic medical chart.
5. When AD is diagnosed, the intervention will be stopped and physicians will carry out appropriate treatment.

### Provisions for Post-trial Care

In case of discontinuation due to illness, we will follow-up participants as long as possible until the situation is restored. If a health issue occurs because of this study, standard treatment will be performed.

### Primary Outcome

Participants will be evaluated for AD at each follow-up visit at 4, 12, 24, and 32 weeks after birth. The primary outcome is the time to the first onset of AD during application of the moisturizer. The time to onset of AD is defined as the number of days between discharge of the newborn and the onset of AD. The UK Working Party's diagnostic criteria (12) will be used as diagnostic criteria of AD.

### Secondary Outcomes

Efficacy endpoints include the following: (1) Eczema Area and Severity Index (EASI) scores (13) at 4, 12, 24, and 32 weeks after birth; (2) patient-oriented eczema measure (POEM) scores (14) during the study; (3) stratum corneum hydration at the date of registration at 4, 12, 24, and 32 weeks after birth; (4) total immunoglobulin E (IgE) antibody titer and specific IgE antibody titer (ImmunoCAP) at the diagnosis of AD or at 32 weeks after birth; and (5) serum thymus and activation-regulated chemokine levels at diagnosis of AD or at 32 weeks after birth (15).

### Safety Outcomes

Safety outcomes describe the frequency and proportion of adverse events.

### Adverse Event Reporting and Harms

In this study, any adverse events that occur during the study intervention period (after discharge) will be investigated, and the investigators will report the following items to the data center: (1) event name; (2) date of onset; (3) severity (mild, moderate, and severe); (4) seriousness (serious, non-serious); (5) intervention measures (continuation, interruption, and discontinuation); (6) measures [none, yes (contents if yes)]; (7) outcomes (recovery, remission, unrecovered, recovered but with sequelae, death, and unknown) and outcome confirmation date; and 8) causal relationship with moisturizer use (can be denied, cannot be denied).

### Participants' Timeline

A summary of participants' timeline is shown in Table 1.

**Table 1:** Assessment schedule.

	**Study period**				**Endpoint**	**Unscheduled medical examination (appearance of skin rash, etc.)**	**When the study intervention is discontinued (including when atopic dermatitis is diagnosed)**
**Visit**	**1**	**2**	**3**	**4**	**5**		
**Time point (visit windows)**	**Entry (6 days old)**	**4 weeks after entry (±7 days)**	**12 weeks after entry (±14 days)**	**24 weeks after entry (±28 days)**	**32 weeks after entry (±28 days)**		
**ENROLLMENT**							
Eligibility screen	X						
Informed consent	X						
Allocation	X						
**ASSESSMENTS**							
Background information	X						
Living environment	X				X		X
Nutrition	X				X		X
Body height	X	X	X	X	X	X	X
Body weight	X	X	X	X	X	X	X
Evaluation of the presence or absence of atopic dermatitis by a blinded physician	X	X	X	X	X	X	X
EASI score by a blinded physician		X	X	X	X		X
POEM score by caregivers		→	→	→	→		→
Stratum corneum hydration	X	X	X	X	X		X
Total IgE, egg white, ovomucoid, milk, wheat, peanut, and *Dermatophagoides farinae*-specific IgE and TARC in serum					X		X
Check the diary		X	X	X	X	X	X
Amount of emollients used		X	X	X	X	X	X
Combination therapy		X	X	X	X	X	X
Adverse events		X	X	X	X	X	X

*EASI, Eczema Area and Severity Index; POEM, patient-oriented eczema measure; TARC, thymus and activation-regulated chemokine; IgE, immunoglobulin E*.

### Sample Size Calculation

A total of 60 participants, with 20 in each group, will be enrolled in this study. This is an exploratory study, and the target case size was selected based on the research period and available resources (feasibility). In our institute, the number of births exceeds ~2,000 per year, and the number of singletons born at 37 weeks after pregnancy is ~1,200 per year. An epidemiological study of AD in Japan showed that the overall AD prevalence was 6.9%. Moreover, the AD prevalence was 9.8, 8.7, 4.4, and 2.6%, respectively, for those in their 20, 30, 40, and 50/60s. The results showed that AD prevalence was higher in women than in men (9.3 vs. 5.1%) (16). Recently, a Japanese national cohort study showed that maternal AD diagnosis was found in 15.7% of mothers (17). Therefore, recruiting 60 neonates within the 8-month registration period should be possible.

### Recruitment

We will place posters and pamphlets around the examination room at our institute. We will perform provisional enrollment and make a list if parents are willing to participate in the study and the offspring may meet the inclusion criteria. When a tentatively listed pregnant woman gives birth, we will reconfirm that the selection criteria are met and recruit the woman.

### Assignment of Interventions: Allocation

#### Sequence Generation

Participants will be randomly assigned in a 1:1:1 to groups A, B, and C using permuted block randomization. A computer-generated allocation sequence will be created by the study statistician using SAS PROC PLAN. The block size is masked from all except the statistician to ensure allocation concealment.

#### Concealment Mechanism/Implementation

We will use VIEDOC4 (Pharma Consulting Group Japan K.K., Tokyo, Japan) for enrollment for this study, and will enroll the subjects and randomize them. The participant's identification number and the assigned group will be displayed on electronic data capture, and a registration confirmation notification will be automatically sent to the study physicians and the data center by email. The study physicians will print and store the registration confirmation notification email.

### Assignment of Interventions: Blinding

#### Who Will Be Blinded

This study separates outpatient physicians from skin-assessment physicians. The physicians who measure EASI and diagnose AD (i.e., evaluate only the skin and do nothing else) will be blinded to the group allocations and will not look at documents and any other information about the study intervention. We will also instruct participants not to reveal which group they are assigned to when they have a skin evaluation. The two moisturizers are both colorless and odorless. A separate person will assess the amount of remaining topical product to determine how much product was applied.

### Data Collection and Management

#### Plans for Assessment and Collection of Outcomes

A summary of the data collection plan is shown in Table 1. Background information, the living environment, and nutrition will be provided by interview. Adherence and POEM scores will be obtained from the diary. The EASI score and the diagnosis of AD will be obtained via physical examination by a blinded physician.

### Data Management

The electronic data capture system will be used for data in this study. All data management will be performed independently by the data center of the NCCHD.

### Confidentiality

During conducting research, the staff involved in the study will establish safety management and systems to protect personal information. All data of the participants who withdrew the consent will be discarded; however, the other data will be stored in VIEDOC infrastructure according to “methods in analysis to handle protocol non-adherence and any statistical methods to handle missing data,” which is hosted in data centers with state-of-the-art surveillance and access control, redundant internet and power feed, and protection against theft, fire, and natural disasters. The data of the participants will be used for the phase III study.

### Plans for Collection, Laboratory Evaluation, and Storage of Biological Specimens for Genetic or Molecular Analysis in This Trial/Future Use

The study physicians will properly store the samples obtained in this study until 5 years after the study is completed.

### Statistical Methods

#### Statistical Methods for Outcomes

Analysis will be performed according to the intention-to-treat principle. To assess whether group A or group B is more effective than group C in preventing the onset of AD, we will use two co-primary analyses using the log-rank test for the time to the onset of AD. The significance level of each comparison is 2.5%. For each group, Kaplan–Meier estimates of the AD-free rate will be determined. Additionally, the Cox proportional hazards model will be used to estimate the hazard ratios of groups A vs. C and groups B vs. C, and their 95% CIs. The CIs will be calculated using the Wald type method. The exact method will be applied to adjust for tied observation times. A test with Schoenfeld residuals will be used to assess the proportional hazard assumption. If a significance level of 10% rejects the validity of the proportional hazards assumption, group comparisons using the difference in the restricted mean survival time will be applied as a supplementary analysis of the co-primary analysis. For the secondary endpoints, the mean and standard deviation of continuous variables, and the frequency and proportion of discrete variables will be calculated for each treatment group and time point. For the safety endpoints, the frequency and proportion of adverse events occurring will be calculated for each treatment group.

#### Interim Analyses

We do not plan an interim analysis.

#### Methods for Additional Analyses (e.g., Subgroup Analyses)

We do not plan additional analyses.

#### Methods in Analysis to Handle Protocol Non-adherence and any Statistical Methods to Handle Missing Data

Analysis of the primary and secondary outcomes will be performed on the full analysis set. As a reference, analyses will be performed for each protocol set.

(1) Full analysis set

Among all randomized subjects, the following will be excluded:

1. Subjects who have never had study intervention
2. Subjects for whom data were not obtained since the start of the study intervention
3. Subjects found to be in violation of eligibility criteria after the fact

(2) Per protocol set

A group of subjects in the full analysis set who do not have significant deviation from the implementation plan will be included. Subjects with the following will be excluded from this group:

1. Poor adherence (application amount is <70% of the planned application amount during the study intervention period, or the number of days that the correct number of applications can be performed is <70% of the planned application period during the study intervention period)
2. If adherence cannot be evaluated
3. When using bath salts for longer than 7 days
4. Moisturizer other than the test moisturizer or control moisturizer, skin external medicines, and skin application agents are applied for 7 days or longer (excluding external use only around the mouth, pubic area, and anus)

#### Plans to Provide Access to the Full Protocol, Participant Level-Data, and Statistical Code

We do not plan to provide access to the full protocol, participant level-data, and statistical code.

#### Oversight and Monitoring

##### Composition of the Coordinating Center and Trial Steering Committee

The coordinating center is located in the Allergy Center, NCCHD. YI is the principle investigator. KYH will coordinate the PAF study. KP is a biostatistician who will analyze the data. TM and YO will advise the study process.

##### Composition of the Data Monitoring Committee, and Its Role and Reporting Structure

Central and onsite monitoring will be conducted to ensure that this study is conducted safely and according to the research protocol and that data are being collected accurately. Monitoring will be conducted by a person designated by the investigator. The study is independent from the sponsor and competing interests. An independent data monitoring committee will be established in this study to monitor the safety of the study.

##### Frequency and Plans for Auditing Conduct of the Trial

We do not plan auditing in this study.

##### Plans for Communicating Important Protocol Amendments to Relevant Parties (e.g., Trial Participants and Ethical Committees)

After the approved Clinical Research Review Committee at this facility approves a change in the research implementation plan, notification of this change will be submitted to the local welfare department. After approval by the accredited clinical research review board, approval of the administrator of the implementing medical institution regarding the revised content will be obtained. If permission is obtained, the investigators will send a copy of the permit to the Research Secretariat and changes to the research protocol will take effect.

## Discussion

This is the first phase II, three-armed RCT in Japan to estimate how effective the twice daily or once daily application of Fam's Baby moisturizer is for preventing AD compared to the once daily application of 2e moisturizer, which was suggested to have a preventive effect on AD in neonates with a high-risk of developing AD. In this study, we will use 2e once daily as a control moisturizer, which we confirmed as effective for primary prevention of AD in our previous RCT (9). Based on the results of this study, we hope that a phase III study will be conducted to determine the optimal method for preventing AD *via* moisturizer application.

Filaggrin (FLG) mutation carriers have an increased risk of AD (18). Because participants have an AD family history, it is presumed that the rate of participants with FLG mutations is relatively high, and children with FLG mutations may have a higher chance of preventing AD with the use of moisturizers. FLG mutations, this time, are not evaluated because of research costs and feasibility. However, we believe that randomizing participants controls the variation between FLG mutation groups and minimizes the impact on results. In phase III, we would like to evaluate the differences in effect depending on the presence or absence of FLG mutation.

### Other Similar Studies

As mentioned above, our previous RCT provided evidence that protecting the skin barrier with a moisturizer applied at the beginning of the neonatal period prevented development of infantile AD (9). However, prevention of AD was not proven in the study (10) in high-risk neonates in the UK and in the study (11) in general populations of Norway and Sweden. Once daily moisturizer was proposed in the study protocols of these two previous studies (10, 11). Reasons for the inconsistency in results for moisturizer interventions might be due to different countries and cultures, different types of moisturizers, and differences in adherence. Dissanayake et al. (19) performed an RCT in Japan to examine whether combined synbiotics and skin moisturizers could prevent AD and food allergy. However, they did not show efficacy of moisturizer application two to three times/day against prevention of AD and food allergy. The actual amount of moisturizer application and adherence in their study were unclear. Lowe et al. (20) reported that applying a sufficient amount of moisturizer twice daily (≥5 days per week, good adherence) in neonates was preventable for sensitization of IgE at the age of 12 months in a small pilot RCT. We believe that the key points of a strategy for preventing allergies by skin moisturizer are as follows: (1) applying a sufficient amount of moisturizer, (2) twice daily application of moisturizer, and (3) the quality of the moisturizer. An individual patient data meta-analysis is ongoing by an international collaboration group (SCiPAD) to identify whether skin care interventions in infants prevent development of AD and food allergy (21). New evidence for skin care prevention, such as application of moisturizer, will be determined.

### Potential Benefits of the Study

If we establish a novel strategy for preventing development of AD by this skin moisturizer intervention for high-risk neonates, the prevalence of AD is expected to decrease and medical care costs will be reduced.

### Dissemination Plans

Main research results will be submitted to academic journals after the final analysis. The co-authors will be decided in accordance with the Uniform Requirements for Manuscripts Submitted to Biomedical Journals of the International Committee of Medical Journal Editors.

### Trial Status

Data are currently being collected. The protocol version number is 1.5 and the date is 1 April 2021. Recruiting participants began at 21 August 2020, and completed by September 2021.

## Ethics Statement

The studies involving human participants were reviewed and approved by the Accredited Clinical Research Review Committee of the National Center for Child Health and Development. Written informed consent to participate in this study was provided by the participants' legal guardian/next of kin.

## Author Contributions

YI is the principle investigator of the PAF study and wrote the first draft of the manuscript. KP is the study statistician. All authors contributed to conception and design of the study and have read and approved the final manuscript.

## Funding

This study received funding from Fam's Inc. (Tokyo, Japan). Clinical nursing support is provided by the Clinical Center of NCCHD. The funder was not involved in the study design, collection, analysis, interpretation of data, the writing of this article or the decision to submit it for publication.

## Conflict of Interest

This trial is conducted on the basis of a collaboration between the NCCHD and Fam's Inc. YO received funding from Fam's Inc. Fam's Inc. will not be involved in the collection, analysis, interpretation of data, or decision to submit results. The remaining authors declare that the research was conducted in the absence of any commercial or financial relationships that could be construed as a potential conflict of interest.

## Publisher's Note

All claims expressed in this article are solely those of the authors and do not necessarily represent those of their affiliated organizations, or those of the publisher, the editors and the reviewers. Any product that may be evaluated in this article, or claim that may be made by its manufacturer, is not guaranteed or endorsed by the publisher.

## Abbreviations

AD: atopic dermatitis
CI: confidence interval
EASI: Eczema Area and Severity Index
IgE: immunoglobulin E
NCCHD: National Center for Child Health and Development
PAF: prevention of atopic dermatitis by applying Fam's Baby
POEM: patient-oriented eczema measure
RCT, randomized: controlled trial
TARC: thymus and activation-regulated chemokine.
